# Transcranial Doppler Use in Non-traumatic Critically Ill Children: A Multicentre Descriptive Study

**DOI:** 10.3389/fped.2021.609175

**Published:** 2021-07-02

**Authors:** Virginie Rollet-Cohen, Philippe Sachs, Pierre-Louis Léger, Zied Merchaoui, Jérôme Rambaud, Laureline Berteloot, Manoëlle Kossorotoff, Guillaume Mortamet, Stéphane Dauger, Pierre Tissieres, Sylvain Renolleau, Mehdi Oualha

**Affiliations:** ^1^Paediatric Intensive Care Unit, Necker-Enfants Malades University Hospital, Assistance Publique des Hôpitaux de Paris, Paris, France; ^2^Paediatric Intensive Care Unit, Robert Debré University Hospital, Assistance Publique des Hôpitaux de Paris, Paris, France; ^3^Paediatric and Neonatal Intensive Care Unit, Trousseau University Hospital, Assistance Publique des Hôpitaux de Paris, Paris, France; ^4^Pediatric Intensive Care, Paris South University Hospital, Assistance Publique Hôpitaux de Paris, Le Kremlin Bicêtre, Paris, France; ^5^Paediatric Radiology Department, Necker-Enfants Malades University Hospital, Assistance Publique des Hôpitaux de Paris, Paris, France; ^6^Paediatric Neurology Department, French Centre for Paediatric Stroke, Necker-Enfants-Malades University Hospital, Assistance Publique des Hôpitaux de Paris, Paris, France; ^7^Paediatric Intensive Care Unit, Grenoble University Hospital, Grenoble, France

**Keywords:** doppler ultrasonography, transcranial, pediatric intensive care unit, critically ill children, nervous system diseases, neuro monitoring

## Abstract

**Background:** The use and perceived value of transcranial Doppler (TCD) scope in paediatric critical care medicine has not been extensively documented.

**Objective:** To describe the use of TCD to assess non-traumatic brain injury in patients admitted to four paediatric intensive care units (PICUs) in France.

**Methods:** We prospectively included all children (aged under 18) assessed with inpatient TCD between November 2014 and October 2015 at one of the four PICUs. The physicians completed a questionnaire within 4 h of performing TCD.

**Results:** 152 children were included. The primary diagnosis was neurological disease in 106 patients (70%), including post ischemic-anoxic brain insult (*n* = 42, 28%), status epilepticus (*n* = 19, 13%), and central nervous system infection/inflammation (*n* = 15, 10%). TCD was the first-line neuromonitoring assessment in 110 patients (72%) and was performed within 24 h of admission in 112 patients (74%). The most common indications for TCD were the routine monitoring of neurological disorders (*n* = 85, 56%) and the detection of asymptomatic neurological disorders (*n* = 37, 24). Concordance between the operator's interpretation of TCD and the published normative values was observed for 21 of the 75 (28%) TCD abnormal findings according to the published normative values. The physicians considered that TCD was of value for the ongoing clinical management of 131 (86%) of the 152 patients.

**Conclusion:** TCD is commonly used in French PICUs and tends to be performed early after admission on patients with a broad range of diseases. The physicians reported that the TCD findings often helped their clinical decision making. In view of the subjectivity of bedside interpretation, true TCD contribution to clinical care remains to be determined. Objective studies of the impact of TCD on patient management and clinical outcomes are therefore warranted.

## Introduction

Brain injury and other neurological conditions are frequently encountered in paediatric intensive care units (PICUs) and constitute the most common proximate causes of death in the children admitted to these units (1, 2). The management of neurological disorders is challenging in critically ill children, since sedation often limits daily clinical evaluations. Transcranial Doppler (TCD) ultrasound enables the bedside evaluation of these patients; it is a non-invasive, readily available technique for the real-time assessment and monitoring of cerebral blood flow.

There is a robust body of literature data on the value of TCD in critically ill adults with various acute neurological conditions. TCD shows high sensitivity and specificity for the diagnosis of vasospasm after subarachnoid haemorrhage, acute middle cerebral artery (MCA) occlusions and brain death (3–5). Furthermore, TCD is a reliable method for estimating the intracranial pressure (ICP) - particularly in the context of traumatic brain injury (6–8). The use of TCD has also been described in the management of sepsis (9), central nervous system (CNS) infections (10, 11), and liver and kidney failure (12–14) – all conditions that are frequently managed in intensive care units (ICUs).

At present, there is good evidence to support the use of TCD in screening for sickle cell disease in children and whose risk of a first stroke would be reduced by a blood transfusion (15, 16). Recently, PICU intensivists have become increasingly interested in the broader application of TCD, given its convenience and proven diagnostic and prognostic value in specific adult populations (17, 18). However, the inherent difficulties of PICU-based research (including frail patients and small study populations) mean that robust data on the influence of TCD use on PICU patient outcomes are scarce. TCD is therefore not currently recommended by the international guidelines on paediatric neurocritical care. However, the results of mostly observational studies and case reports suggest that TCD can provide relevant cerebrovascular haemodynamic measurements in children with various neurological conditions (17–19), including stroke and cerebrovascular disorders (20–22), CNS infections (23–28), and brain death (29–31). TCD may also be applicable to conditions other than primary neurological dysfunctions, such as the assessment of children with diabetic ketoacidosis (32, 33) and those on extracorporeal membrane oxygenation (ECMO) (34–36).

The frequency and timing of TCD use, the indications for investigation, and the influence of TCD findings on clinical practice in the PICU have been rarely described - especially in Europe. An international, web-based survey found that TCD was frequently used in North American neurocritical care centres and was considered as a useful guide for patient management (37). The primary objective of the present prospective, multicentre study was to describe the scope of TCD use (notably with respect to the technique's timing and clinical indications) in four French PICUs. The secondary objective was to evaluate the physicians' opinion of the utility of TCD in routine patient management.

## Methods

### Study Design and Population

This was a prospective, descriptive, multicentre study of all children (aged <18) having undergone an assessment with TCD between November 2014 and October 2015 at one of four French PICUs (Necker Hospital, Paris; Trousseau Hospital, Paris; Robert Debré Hospital, Paris; Kremlin Bicêtre Hospital, Le Kremlin Bicêtre). Only data from the first TCD measurement in each patient were evaluated. Patients with traumatic brain injury, incompletely reported TCD values, TCD measurement failure or missing clinical data in the questionnaire were excluded. TCD use and patient care was at the discretion of the treating intensivist. All intensivists were senior specialists and had at least 6 months of experience with TCD. Younger operators were supervised by a senior intensivist until they were deemed completely autonomous. The study was approved by an independent ethics committee and performed in accordance with the tenets of the Declaration of Helsinki. Informed consent was obtained from all subjects or their legal guardian.

### Data Collection

TCD was performed using a commercially available unit (Vivid S5 ultrasound, General Electric Medical Systems). The depth and angle of insonation that gave the highest mean flow velocity and best waveform measurements were selected. Each operator completed a questionnaire (see the Supplementary Material) within the 4 h of the TCD session. The questionnaire included questions on (i) the operator's level of training (<1 or ≥1 year of regular TCD practice in a PICU), (ii) the patient characteristics (clinical characteristics, neurological findings from a physical examination and from previous neurological examinations), and (iii) characteristics of the TCD (technical conditions, timing, indications, suspected abnormalities, TCD values and patterns, interpretation, and the physician's opinion on the utility of TCD in diagnostic and therapeutic management).

With regard to the criteria used for TCD abnormal findings in various patient groups, the intensivist were provided with tables specifying normative data for TCD measurements of the MCA as a function of the patient's age, sex, and sedation conditions (38–41).

Values of peak systolic flow velocity (PSV) and end diastolic flow velocity (EDV) were extracted via the questionnaire. Similarly, the mean flow velocity (MFV), pulsatility index (PI), and resistivity index (RI) were extracted via the questionnaire or (if not available) were calculated using previously published equations (42). Potent determinants of blood flow velocities were recorded as follows, with the normative values shown in brackets: temperature (36°C−37.5°C), oxygen saturation (>92%), venous PCO_2_ (40–50 mmHg), arterial PCO_2_ (35–45 mmHg) and/or end-tidal PCO_2_ (31–41 mmHg). Arterial blood pressures, heart rates and haemoglobin levels were interpreted as a function of the patient's age (43, 44). Other clinical data (including the primary diagnosis, the neurological diagnosis and the patient outcomes) were extracted from the patient's electronic medical records.

The severity of critical illness was assessed in terms of the Pediatric Logistic Organ Dysfunction (PELOD) score on the day of the TCD evaluation (45). Organ dysfunction was defined according to the latest international consensus conference on paediatric sepsis (43). Primary and neurological diagnoses were defined according to the International Classification of Diseases, 11th edition (ICD-11).

The following therapeutic interventions were reported: (i) haemodynamic optimization (fluid resuscitation or administration of vasopressors/inotropes), (ii) the de-escalation (discontinuation or dose reduction) of neurological drug treatments such as osmotherapeutic agents, sedatives, and neuromuscular blocking agents, and (iii) the escalation (initiation, addition or intensification) of the above-mentioned neurological drug treatments.

### Data Analysis

Descriptive statistics were calculated and expressed as the median [interquartile range (IQR)] or the range for continuous variables and the frequency (percentage) for binary or categorical data. All intergroup comparisons of binary or categorical variables were performed with a chi-squared test, while intergroup comparisons of continuous variables were assessed with the non-parametric Mann–Whitney U test. Statistical correlations were investigated by calculating Pearson's coefficient. The threshold for statistical significance was set to *p* < 0.05 in all cases.

## Results

Of the 2,961 patients admitted to the four PICUs between November 2014 and October 2015, 198 (6.7%) underwent a total of 324 TCD assessments. After the exclusion of 46 cases (missing TCD values, *n* = 25; measurement failure, *n* = 10; missing clinical data, *n* = 6; patients with traumatic brain injuries, *n* = 5), 152 patients were included in our analysis. Forty-three patients had 2 or more TCD assessments during their stay in the PICU.

### Patient Characteristics

The characteristics of the study population (*n* = 152) are summarized in Table 1. The median (range) age was 7.6 months (0–206). There were 38 (25%) neonates (under 28 days of age), 63 infants (42%) between 28 days and 2 years of age, 41 children (27%) between 2 and 10 years of age, and 10 children (6%) over the age of 10. Twenty patients (13%) were premature infants. Twenty-two patients (14%) had a pre-existing neurological comorbidity (mainly disabilities: *n* = 16). Sixty-nine patients (45%) had other comorbidities, mainly immune/haematological disorders (*n* = 15, 10%), or cardiovascular disease (*n* = 14, 9%). One hundred and thirty-nine (91%) patients presented with organ dysfunction (Table 1). Fifty-seven children had concomitant dysfunction of at least three organ systems.

**Table 1:** Characteristics of the study population.

**Variables**	***n* (%) or median [IQR]**
Population study	152 (100)
**Demographics**	
Age (months)	7.6 [0.9–43]
Sex ratio (M/F)	1.1 (78/74)
Comorbidities	79 (52)
PELOD score	11 [2–23]
**Main reason for admission**	
Neurological disorder	102 (67)
Haemodynamic disorder	21 (14)
Respiratory disorder	16 (11)
Other disorder	13 (8)
**Organ dysfunction**	
Neurological	113 (74)
Respiratory	92 (61)
Haemodynamic	54 (36)
Other	61 (40)
No organ dysfunction	13 (9)
**Therapies**	
Mechanical ventilation	117 (77)
Sedative drugs	107 (70)
Neuromuscular blocking agents	33 (22)
Vasopressors	40 (26)
ECMO	21 (14)
CRRT	3 (2)
**Neurological examination findings**	
Coma	85 (56)
Pupils	
abnormal	45 (30)
normal	96 (63)
missing data	11 (7)
Oculomotricity	
abnormal	3 (2)
normal	62 (41)
missing data	87 (57)
Focal sign	
yes	9 (6)
no	90 (59)
missing data	53 (35)

*IQR, interquartile range; PELOD, pediatric logistic organ dysfunction; ECMO, extracorporeal membrane oxygenation; CRRT, continuous renal replacement therapy; M, male; F, female*.

**Figure 1:**
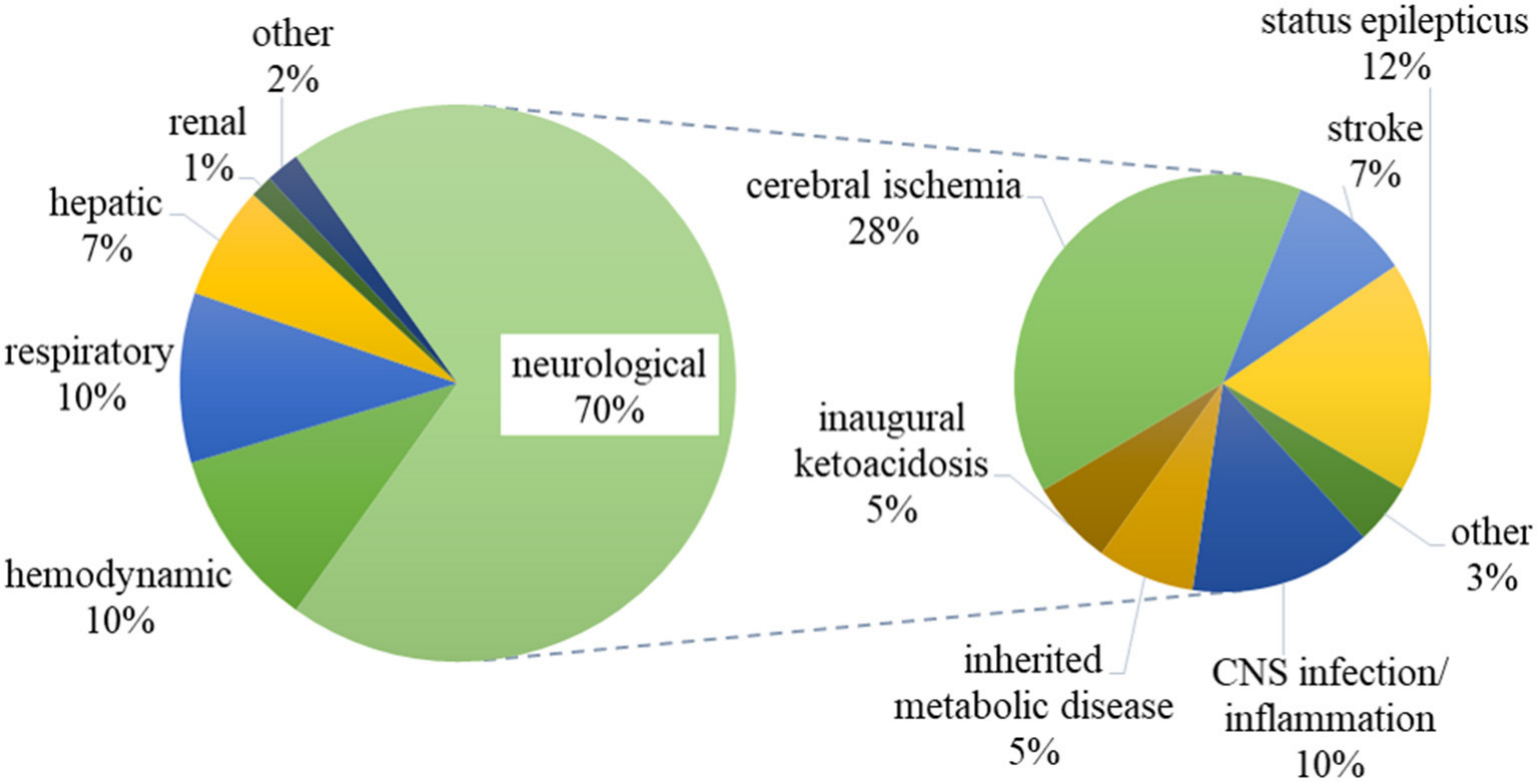
Primary diagnoses of the patients included in the study. CNS: central nervous system.

The PICUs' primary diagnoses are summarized in Figure 1. The majority of the primary diagnoses corresponded to neurological diseases (*n* = 106, 70%), over half of which were due to post-ischemic/anoxic brain injury (*n* = 42, including 19 cases of neonatal ischemic hypoxic encephalopathy), a CNS infection or inflammation (*n* = 15), or status epilepticus (*n* = 19). Acute respiratory distress syndrome and acute lung injury were the most common respiratory diagnoses. The main haemodynamic diagnoses were cardiogenic and septic shock. The median (range) length of stay in the PICU was 7 days (1–84). A total of 32 (21%) patients died during their stay in the PICU – mostly from neurological causes (*n* = 20).

### Characteristics of the TCD

#### Operators

TCD was performed by 43 different operators, of whom 21 had more than 1 year of PICU-based TCD experience. These 21 operators performed 103 of the TCD assessments (68%) analyzed here.

#### Technical Conditions

The intensivists considered that the technical conditions were easy for 114 of the TCD assessments (75%) and difficult for 5 (0.3%; missing data, *n* = 33). The temporal window was mostly used (*n* = 134, 88%), and the MCA was the most frequently recorded vessel (*n* = 143, 94%). The cerebral artery signal was recorded at a mean depth of 3.8 cm.

#### Indications and Timing for TCD Assessments

The main indications for TCD were the routine monitoring of neurological disorders (n=85, 56%), screening for asymptomatic neurological disorders (*n* = 37, 24%), and the evaluation of the onset or the worsening of neurological disorders 17% (*n* = 26) (Table 2). More than one indication were reported for 23 TCDs (15%). Before the TCD assessment, elevated PI was suspected in 80 patients (53%) (Table 2). Several TCD abnormalities were suspected in 70 patients (46%).

**Table 2:** Characteristics of the TCD assessments: indications and suspected abnormalities.

**Variables**	**n (%)**
**Indications**	
Neurological disorder monitoring	85 (56)
Detection of asymptomatic neurological disorder	37 (24)
Onset/worsening of neurological disorder	26 (17)
Haemodynamic disorder	14 (9)
Others	14 (9)
**Suspected TCD abnormalities**	
Elevated PI	80 (53)
Cerebral hypoperfusion	39 (26)
Cerebral hyperaemia	33 (22)
Asymmetric perfusion	31 (20)
Reverse flow	11 (7)
Not documented	4 (3)

*TCD, transcranial Doppler; PI, pulsatility index*.

One hundred and twelve TCDs (74%) were performed within 24 h of admission to the PICU. The median (range) time interval between the onset of the neurological disorder and the TCD was 12 h (1.5–96). One hundred and four TCDs (68%) were performed during night or weekend shifts.

TCD was performed prior to neuroimaging (CT and/or brain MRI) in 110 patients (72%). Neuroimaging was performed prior to TCD in 42 patients (CT: *n* = 38; MRI: *n* = 15; CT and MRI: *n* = 11). Electroencephalography and lumbar puncture were performed before TCD in 55 and 6 patients, respectively.

#### TCD Findings

The TCD findings were considered to be abnormal by the operator in 78 patients (51%) and normal in 68 (45%). The operator could not form an opinion in 6 cases (4%). The main abnormal findings according to the operator were hyperaemia (*n* = 35), hypoperfusion (*n* = 17), asymmetric perfusion (*n* = 16), elevated IP (*n* = 10), and reverse flow (*n* = 4).

The median values obtained from TCD measurements of the MCA (*n* = 143) and potent systemic determinants of cerebral blood flow velocities are presented by patient age in Supplementary Tables 1, 2, respectively. These determinants were all normal in 21 patients (14%).

The values of TCD variables for the MCA for TCD assessments considered to be “normal” vs. “abnormal” are shown in Supplementary Table 3. These two subgroups did not differ significantly, except for the median MFV in the neonates (32 cm/s vs. 25 cm/s for normal and abnormal findings, respectively; *p* = 0.02) and the PI in children between 28 days and 2 years (1.1 vs. 1.4, respectively; *p* = 0.01).

We also analyzed the level of agreement between the operator's interpretation and the published normative values (Table 3). Concordance between the two was observed for 21 of the 75 (28%) TCD abnormal findings according to the published normative values. The concordance rates for the various TCD abnormalities were 67% for reverse flow, 50% for elevated PI, 38% for hyperaemia, 22% for hypoperfusion, and 22% for asymmetry. Forty-three percentage of the TCD assessments were considered to be normal by both the operators and with regard to the normative values.

**Table 3:** Concordance between TCD diagnoses according to operator vs. published normative values.

	**According to the normative values**								
**According to the operator**		**Hyperemia**	**Hypoperf**.	**Asymmetry**	**Elevated PI**	**Reverse flow**	**Normal**	**ND**	**Total**
	Hyperemia	**6 (38%)**	1	10	0	0	8	10	35
	Hypoperf.	0	**2 (16%)**	4	2	1	2	6	16
	Asymmetry	1	1	**8 (22%)**	1	0	5	0	16
	Elevated PI	0	2	2	**3 (43%)**	0	3	0	10
	Reverse flow	0	1	1	0	**2 (67%)**	0	0	4
	Normal	8	4	10	0	0	**15 (43%)**	31	68
	ND	1	1	2	1	0	2	2	9
	Total	16	12	37	7	3	35	43	

*Concordance rates are given in brackets. Hypoperf: hypoperfusion. PI: pulsatility index. ND: not documented. Each TCD parameter was defined as low (below 2 standard deviations, SD), normal, or high (greater than 2 standard deviations) according to the literature data (38–41)*.

### The Intensivists' Opinion of the Utility of TCD

According to the intensivists, TCD made a useful contribution to the clinical management of 131 patients (86%). A contribution to diagnosis was reported for 109 patients (72%). TCD confirmed the main suspected abnormality in 28 patients, reinforced it in 16 patients, made it less likely in 25 patients, and ruled it out in 37 patients (missing data, *n* = 3). For patients diagnosed with neurological disorders, TCD strengthened the suspected diagnosis in 15 cases and made it less likely in 15 other cases. For two patients, TCD suggested a diagnosis that was not initially considered by the intensivist (brain haemorrhage, and vasoconstriction due to hypocapnia).

A useful contribution to therapeutic management was reported for 55 patients (36%). TCD-instigated therapeutic interventions included haemodynamic optimization (*n* = 27, 49%), neuroprotective strategies (intubation, sedation, and the management of secondary brain injuries of systemic origin) (*n* = 24, 44%), the escalation of neurological treatment (*n* = 7, 13%), specific anti-oedema treatment (*n* = 5, 9%), and the de-escalation of neurological treatment (*n* = 4, 7%).

There were no demographic or clinical differences between the groups of patients with *vs*. without therapeutic intervention. Therapeutic intervention was significantly associated with an “abnormal” TCD assessment, according to the operator (*n* = 42 out of 55 (76%), vs. *n* = 35 out of 97 (36%) “normal” TCD assessments; *p* < 0.001; Supplementary Table 4).

The use of TCD prompted additional neurological investigations in 14 patients: a CT scan (*n* = 5), MRI (*n* = 4), electroencephalography (*n* = 4), and eye fundus examination (*n* = 1). One TCD assessment identified a contraindication to lumbar puncture.

## Discussion

The data from our prospective multicentre study enabled us to describe the modalities and scope of TCD use in a large cohort of non traumatic critically ill children. The main findings were that (i) TCD was used by intensivists in the management of a broad range of heterogeneous conditions, (ii) TCD was performed early after admission to the PICU, and (iii) the intensivists reported that TCD contributed to the clinical management of the majority of patients.

Our results showed that TCD is used frequently (for 6.7% of patients, on average) in four French PICUs. The study design might have influenced this finding by inciting intensivists to perform more TCD assessments than they would have done routinely. However, the prevalence of 6.7% found here is in line with the value reported in a large, European, one-day, observational, multicentre investigation of the use of point-of-care ultrasound in adult and paediatric ICUs (46).

The broad range of clinical indications for TCD [encompassing almost all diseases in critically ill children for which the use of TCD has ever been described (18)] was an important finding of the present study. The recent web-based survey by LaRovere et al. also reported that TCD was used to investigate a dozen or so conditions in clinical practice (37). There is great scope for TCD use beyond the evaluation of patients with primary neurological disorders. This notably included patients at risk of neurological damage (i.e., sepsis or diabetic ketoacidosis), as highlighted in our study by the proportion of patients without an initial neurological dysfunction (26%) investigated with TCD. Furthermore, TCD was used to detect asymptomatic neurological disorders (*n* = 37, 24%) and monitor haemodynamic disorders (*n* = 14, 9%). Lastly, TCD was used to monitor cerebral blood flow in patients having had received ECMO (15% of the cohort), since this intervention can induce severe neurological complications. Our finding is in line with the recently observed trend towards the use of TCD in this indication (34–36).

The early use of TCD (within 24 h of admission), its status as a first-line neuromonitoring tool, and the frequency of use during night shifts highlighted the ready availability of this technique. TCD therefore has the potential to become an influential neuromonitoring strategy in the PICU (37, 47).

According to the surveyed physicians, TCD contributed to the clinical management of the majority of the patients that they examined (86%). The number of TCD assessments performed in our study might have been oversetimated because of the Hawthorne effect. Similarly, the use of TCD was not controlled and was left to the intensivists' discretion, who might already have held a positive view of TCD's contribution. However, our findings were similar to those reported in the web-based survey by La Rovere et al. (37). Of the 27 centres that routinely used TCD, 20 (75%) used the findings to guide clinical care (37). Although both La Rovere et al.'s study and the present study might have been prone to patient selection bias and reporting bias, the latter are unlikely to fully account for the observed results.

Along with TCD's broad range of applications, the major contributions of TCD reported in La Rovere et al.'s study and the present study raise the question of potential misuse. Standardized guidelines on TCD assessments in paediatric patients are still lacking. Leaving the use and interpretation of TCD to the physician's discretion might increase the risk of inappropriate management of critically ill children. The low observed rate of agreement between the operator's judgment on one hand and the normative values on the other emphasized the difficulty and uncertainty of interpreting TCD data outside their clinical context. Indeed, the classification of TCD results as “normal” or “abnormal” was not directly related to the operator-reported values of the TCD variables. It is likely that the values considered to be normal (i.e., according to the published values) are classified as abnormal (and *vice versa*) in the PICU because of the particular clinical situation at a given time in an unstable, critically ill patient. Similarly, the variability in potent systemic determinants of flow velocities might have influenced the interpretation of TCD variables. TCD ultrasound was developed recently and so should be used with caution by taking account of the particular features of each individual situation.

Interestingly, French intensivists perform and interpret TCD data themselves; this contrasts with the situation in the USA, where TCD is mostly performed by neurovascular specialists. The French practice appears to be advantageous for integrating the TCD results into the broader clinical context but also highlights the necessity for ongoing technical and clinical training in TCD and the development of specific TCD training for intensivists. In 2009, the American College of Chest Physicians's Critical Care NetWork partnered with the French Society of Critical Care Medicine to produce a consensus statement on competence in critical care ultrasonography; however, there was no mention of TCD in these guidelines (48). In France, credentials in the use of TCD field can be acquired after completing a training course with an expert radiologist or sonographer. Unfortunately, none of these courses has been specifically designed for paediatric intensivists. Standardized, PICU-specific measurement techniques, reporting formats, data interpretation methods and training formats are now needed. Accurate interpretation requires an understanding of the technical limitations of TCD and thus encompasses ultrasound physics, cerebrovascular anatomy, and the patient's pathophysiological characteristics. Integration of the findings from a clinical examination (age, sex, temperature, and heart rate), laboratory data (haematocrit, PcO2), and clinical features is mandatory. With this mind, standard operating procedures for data acquisition and the documentation of clinical examinations might be useful tools. They would also facilitate the performance of larger observational studies and interventional trials with objective outcome measures; these trials would help to establish a standardized, evidence-based consensus statement for TCD measurements, interpretation, clinical applications and impacts in critically ill children.

Our study had several limitations. Firstly, the classification of TCD indications given in the questionnaire was not appropriate for accurately determining the specific (correct) indications for TCD use. However, the detailed information from the patients' records might enable us to determine whether TCD was carried out in the context of an acute change in the level of consciousness (for non-sedated patients), raised ICP, non-convulsive seizures, a new focal neurological deficit, or worsening haemodynamic status. Further studies of the impact of TCD on clinical management should include patients with the above conditions. Secondly, the participating PICUs were located in teaching hospitals in the Paris region. Consequently, our findings may be not representative of practice throughout France. Furthermore, the descriptive study design means that the impact of TCD on patient management should be interpreted with caution. Causality cannot be implied, and the subjective interpretation of TCD results by physicians may (in addition to the bias related to differences in the level of training) have influenced patient management.

## Conclusion

Our study provide valuable data on the real-life use of TCD in the PICU. TCD is an easy-to-use technique that is increasingly applied to a broad range of heterogeneous medical conditions and might be of great value for patient management. However, in view of the subjectivity of bedside interpretation, its contribution remains to be determined. The results of TCD should therefore be interpreted in the light of the clinical context and the results of other neurological investigations.

Objective studies of the impact of TCD on patient management and clinical outcomes are therefore warranted, with a view to facilitating the development of evidence-based guidelines on TCD use in the PICU.

## Data Availability Statement

The raw data supporting the conclusions of this article will be made available by the authors, without undue reservation.

## Ethics Statement

The studies involving human participants were reviewed and approved by CENEM: ethics committee of Necker Enfants Malades Hospital, Paris, France. Written informed consent to participate in this study was provided by the participants' legal guardian/next of kin.

## Author Contributions

VR-C: study design, analysis of the results, and drafting of the manuscript. PS, PL-L, ZM, JR, GM, SD, PT, and SR: study design, provision of data, and critical comment on the manuscript. LB and MK: study design, and critical comment on the manuscript. MO: study design, analysis of the results, and drafting of the manuscript. All authors contributed to the article and approved the submitted version.

## Acknowledgements

We thank all the intensivists who performed TCD assessments: Drs Aubelle, Merckx, Baudin, Bernardin, Brousse, Bueno, Carlier, Church, de Saint Blanquat, Delbet, Demoulin, Dubois, Ducrocq, Dupic, Durand, Grimaud, Herisse, Hogan, Jean, Julliand, Le Bourgeois, Le Movel, Léger, Lesage, Lévy, Maimouni, Maurice, Merchaoui, Mesnil, Miatello, Morin, Mortamet, Oualha, Peipoch, Pierre, Rambaud, Renolleau, Sieng, and Torterue. We thank Vassil Stevanov and Caroline Elie for assistance with the statistical analysis. Our thanks also go to the secretaries in the PICUs: Corinne Le Gall (Necker Hospital), Sonia Gallais (Trousseau Hospital), Joëlle Colombier (Robert Debré Hospital), and Corinne Schiaretti (Kremlin-Bicêtre Hospital).

## Conflict of Interest

The authors declare that the research was conducted in the absence of any commercial or financial relationships that could be construed as a potential conflict of interest.
