# Supplementary material for: Transcranial Doppler Use in Non-traumatic Critically Ill Children: A Multicentre Descriptive Study
Source: Front Pediatr. 2021 Jul 2;9:609175. doi: 10.3389/fped.2021.609175 (PMC8282928; doi:10.3389/fped.2021.609175)
Supplement: Supplementary file 1 [file Table_1.DOCX]

**Supplementary table 1: Transcranial Doppler parameters recorded at middle cerebral artery (n=143) by patient age**

| **TCD Parameters** | **Age** | | | |  |
| --- | --- | --- | --- | --- | --- |
|  | **0 – 28 d** | **28 d - 2 y** | **2 - 10 y** | **> 10 y** | **Total** |
| N patients (%) | 39 (27) | 55 (38.5) | 40 (28) | 9 (6.5) | 143 (100) |
| n measurements (%) | 78 (27) | 110 (38.5) | 80 (28) | 18 (6.5) | 286 (100) |
| PSV (cm/sec), median [IQR] | 51 [34-71] | 98 [71-131] | 129 [100-153] | 101 [80-133] | 91 [60-132] |
| EDV (cm/sec), median [IQR] | 15 [8-22] | 32 [22-41] | 52 [41-67] | 37 [26-54] | 31 [18-49] |
| MFV (cm/sec), median [IQR] | 27 [17-38] | 52 [43-71] | 80 [62-98] | 66 [44-79] | 51 [34-78] |
| PI, median [IQR] | 1.4 [1.1-1.7] | 1.2 [0.9-1.5] | 0.9 [0.7-1.1] | 1.1 [0.9-1.2] | 1.1 [0.9-1.5] |
| RI, median [IQR] | 0.7 [0.6-0.8] | 0.7 [0.6-0.8] | 0.6 [0.5-0.7] | 0.6 [0.6-0.7] | 0.7 [0.6-0.8] |

TCD, transcranial doppler; d, days; y, years; IQR, Interquartile range, PSV, peak systolic velocity, EDV, end diastolic velocity; MFV, mean flow velocity; PI, pulsatility index; RI, resistivity index.
