# Supplementary material for: Transcranial Doppler Use in Non-traumatic Critically Ill Children: A Multicentre Descriptive Study
Source: Front Pediatr. 2021 Jul 2;9:609175. doi: 10.3389/fped.2021.609175 (PMC8282928; doi:10.3389/fped.2021.609175)
Supplement: Supplementary file 2 [file Table_2.DOCX]

**Supplementary table 2: Values of systemic potent determinants of cerebral flood velocities by patient age**

| **Variables** |  | **Age** | | | |  |
| --- | --- | --- | --- | --- | --- | --- |
|  | **N patients*** | **0 – 28 d** | **28 d - 2 y** | **2 - 10 y** | **> 10 y** | **Total** |
| pCO2 (mmHg), median [IQR] | 131 | 41.5 [33.5-46.5] | 37.5 [30.8-37.4] | 40 [33-49] | 39.5 [35.3-43.4] | 40 [32-46] |
| Hb (g/dL), median [IQR] | 133 | 13.8 [11.4-17.5] | 10 [9-11.2] | 11 [10-12] | 13.1 [12.5-13.8] | 11.2 [9.9-13] |
| MAP (mmHg), median [IQR] | 140 | 50 [49-59] | 62 [53-73] | 72 [64-81] | 73 [67-88.3] | 64 [52-74.3] |
| HR (bpm), median [IQR] | 143 | 115 [94.5-143] | 140 [123-157] | 125 [111-140] | 90 [81-99] | 130 [110-147] |
| Temperature (°C), median [IQR] | 143 | 35.3 [33.5-36.7] | 37 [36-37.4] | 37 [36.5-37.7] | 36.7 [35.8-37.1] | 36.7 [35.4-37.2] |

d, days; y, years; IQR, Interquartile range; pCO2, carbon dioxide pression; Hb, hemoglobin; MAP, mean arterial pressure; HR, heart rate; bpm : beats per minute.*Patients with TCD parameters recorded at middle cerebral artery.
