# Supplementary material for: Transcranial Doppler Use in Non-traumatic Critically Ill Children: A Multicentre Descriptive Study
Source: Front Pediatr. 2021 Jul 2;9:609175. doi: 10.3389/fped.2021.609175 (PMC8282928; doi:10.3389/fped.2021.609175)
Supplement: Supplementary file 3 [file Table_3.DOCX]

| **Age group** | **<28 days**  (n=36) | | | **28 days-2 years**  (n=55) | | | **2-10 years**  (n=37) | | | **>10 years**  (n=9) | | |
| --- | --- | --- | --- | --- | --- | --- | --- | --- | --- | --- | --- | --- |
| **TCD interpretation** | N  n=17 | AbN  n=19 | p value | N  n=23 | AbN n=32 | p value | N  n=19 | AbN n=18 | p value | N  n=4 | AbN n=5 | P value |
| **TCD parameters** |  |  |  |  |  |  |  |  |  |  |  |  |
| PSV (cm/sec),  median (range), | 59  (18-99) | 47  (22-166) | 0.09 | 103  (40-230) | 97  (0-240) | 0.19 | 137  (76-217) | 120  (54-231) | 0.08 | 113  (63-147) | 83  (43-184) | 0.41 |
| EDV (cm/sec), median (range) | 17  (4-41) | 10  (0-42) | 0.09 | 34  (7-69) | 29  (0-126) | 0.07 | 53  (34-92) | 52  (0-124) | 0.52 | 52  (27-62) | 27  (15-153) | 0.08 |
| MFV(cm/sec),  median (range) | 32  (8-64) | 25  (8-89) | **0.02*** | 55  (18-132) | 51  (0-164) | 0.13 | 83  (51-134) | 73  (23-160) | 0.09 | 76  (39-88) | 46  (25-208) | 0.17 |
| PI median (range) | 1.2  (0.7-2.4) | 1.6  (0.4-6.4) | 0.04 | 1.1  (0.8-2) | 1.4  (0.3-4.2) | **0.01*** | 0.9  (0.6-1.3) | 0.9  (0.2-3) | 0.57 | 0.8  (0.5-1.2) | 1.2  (0.6-1.9) | 0.05 |
| RI median (range) | 0.7  (0.5-0.9) | 0.8  (0.4-6.4) | 0.15 | 0.7  (0.5-1) | 0.7  (0.2-1) | 0.1 | 0.6  (0.4-0.7) | 0.5  (0.2-1) | 0.29 | 0.6  (0.4-0.7) | 0.7  (0.4-0.8) | 0.15 |

**Supplementary table S3: Values of TCD parameters by patient age and according to interpretation of TCD.**

*TCD, transcranial doppler; PSV, peak systolic velocity; EDV, end diastolic velocity; MFV, mean flow velocity; PI, pulsatility index; RI, resistivity index; N: normal; abN: abnormal; *: statically significant*
