# Supplementary material for: Transcranial Doppler Use in Non-traumatic Critically Ill Children: A Multicentre Descriptive Study
Source: Front Pediatr. 2021 Jul 2;9:609175. doi: 10.3389/fped.2021.609175 (PMC8282928; doi:10.3389/fped.2021.609175)
Supplement: Supplementary file 4 [file Table_4.DOCX]

| **Variables** | N (%)  or median [IQR] | N (%)  or median [IQR] | N (%)  or median [IQR] |  |
| --- | --- | --- | --- | --- |
|  | Total | With therapeutic intervention | Without therapeutic intervention | p value |
| **Population study** | 152 (100) | 55 (100) | 97 (100) |  |
| **Demographics** |  |  |  |  |
| Age, months | 7.6 [0.9-43] | 9.7 [0.9-44] | 5.8 [0.9-40] | 0.99 |
| Male | 78 (51) | 27 (49) | 51(52) | 0.4 |
| PELOD score | 11 [2-23] | 11 [2-17] | 11 [12-2] | 0.37 |
| **Main reason for admission** |  |  |  |  |
| Neurological | 102 (67) | 38 (69) | 64 (66) | 0.72 |
| Haemodynamic | 21 (14) | 9 (16) | 12 (12.5) | 0.63 |
| Respiratory | 16 (11) | 4 (7) | 12 (12.5) | 0.77 |
| Other | 13 (8) | 4 (7) | 9 (9) | 0.42 |
| **Therapies** |  |  |  |  |
| Mechanical ventilation | 117 (77) | 44 (80) | 73 (75) | 0.55 |
| Vasopressors | 40 (26) | 15 (27) | 25 (25) | 0.85 |
| ECMO | 21 (14) | 8 (14) | 13 (13) | 1 |
| CRRT | 3 (2) | 0 (0) | 3 (3) | 0.55 |
| **Neurological exam findings** |  |  |  |  |
| Pupils abnormality | 45 (30) | 18 (32) | 27 (28) | 0.58 |
| Focal sign | 9 (6) | 3 (5) | 6 (6) | 1 |
| **DTC interpretation**  Abnormal | 77 (50) | 42 (76) | 35 (36) | **<0.001*** |

**Supplementary table S4:**

**Comparison of groups of patients with *vs* without therapeutic intervention**

*IQR: interquartile range; PELOD: paediatric logistic organ dysfunction; ECMO: extracorporeal membrane oxygenation; CRRT: continuous renal replacement therapy.*: statistically significant.*
