# Supplementary material for: Transcranial Doppler Use in Non-traumatic Critically Ill Children: A Multicentre Descriptive Study
Source: Front Pediatr. 2021 Jul 2;9:609175. doi: 10.3389/fped.2021.609175 (PMC8282928; doi:10.3389/fped.2021.609175)
Supplement: Supplementary file 5 [file Table_5.DOCX]

**NEWBORN (< 28 d)** 🞏

**PRETERM** 🞏

**CHILDREN** 🞏

**PREMATURE (<37SA)** □

**Operator.**  **senior** 🞏  **junior** 🞏

**Experience of TCD : < 1y** 🞏 **1-2 y** 🞏 **> 2 y** 🞏

**Date and hour of completion :**  _____/_____/______ at_______h_______

**Admission motive in PICU :** _____________________________________________________

**A : Informations during examination**

**1. Clinical status**

Sedation: Yes 🞏 No 🞏 ; Neuromuscular blocking adgents: Yes 🞏 No 🞏 ; Intubation :Yes 🞏 No 🞏

Glasgow Coma Scale: ____/15 ; not assessable 🞏

Consciousness alteration (if not sedated): Yes 🞏 No 🞏

Corneal reflex: normal 🞏 abnormal 🞏 not assessable or researched 🞏

*If abnormal*: symmetric 🞏 asymmetric 🞏 (pathologic side: R 🞏 L 🞏)

Pupils: normal 🞏 abnormal 🞏 not assessable or researched 🞏

*If abnormal*: symmetric 🞏 asymmetric 🞏 (pathologic side: R 🞏 L 🞏)

myosis 🞏 mydriasis 🞏 middle 🞏

reactive 🞏 unreactive 🞏

Oculomotor abnormality: Yes 🞏 No 🞏  not assessable or researched 🞏

*If yes* symmetric 🞏 asymmetric 🞏 (pathologic side: R 🞏 L 🞏)

Lability : Heart rate 🞏 Arterial pressure 🞏 Respiratory frequency 🞏 No 🞏

Focal sign : Yes 🞏 No 🞏 not assessable or researched 🞏

*If yes*, specify ________________________________________________________

Fontanel : normal 🞏 abnormal (bulging) 🞏  closed 🞏

Meningeal syndrome : Yes 🞏 No 🞏  not assessable 🞏

Seizures : Yes 🞏 No 🞏 Doubt 🞏 not assessable or researched 🞏

*If yes or doubt* : generalized seizures 🞏 focal seizures 🞏 (seizure side : R 🞏 /L 🞏)

**2. Previous neurological investigations :**

EEG 🞏 SEP 🞏 CT 🞏 MRI 🞏 Fundus 🞏 Lumbar puncture 🞏 Previous TCD 🞏

Ionogram 🞏 Ammoniac 🞏 Toxic 🞏 None 🞏

**3. TCD indication** *(possible multiple answers) :*

Monitoring of neurological disorder 🞏 Onset/worsening of neurological disorder 🞏 Detection of asymptomatic neurological disorder 🞏  Abnormal exam 🞏 Hemodynamic disorder 🞏 Others : ______________

**4.** **Suspected TCD abnormaility (ies) before assessment** :

If several answers, circle the main suspected abnormality :

Reverse-flow 🞏 Cerebral hypoperfusion 🞏 Elevated PI 🞏 Asymmetric perfusion 🞏

Cerebral hyperperfusion 🞏 Others 🞏 (specify)_________________________________

**5. Neurological diagnosis suspected or confirmed before TCD**

Meningitis 🞏 Encephalitis 🞏 Cerebral abscess 🞏 Hydrocephalus 🞏 Thrombophlebitis 🞏

Stroke 🞏 Status epilepticus 🞏 Cerebral ischaemia 🞏 Diabetic ketoacidosis 🞏 hemolytic-uremic syndrome 🞏

Brain death 🞏 Intracranial hypertension 🞏 Brain Edema 🞏 Others 🞏 (specify)_____

6. **TCD’s conditions** good 🞏 poor 🞏

Vasopressors 🞏, Cardiac ultrasound 🞏, NIRS 🞏, Therapeutic hypothermia 🞏

| Temperature (°C) | Mean blood pressure (mmHg) | Heart rate (bpm) | Oxygen saturation level | Capnia | Hemoglobin (g/dL) |
| --- | --- | --- | --- | --- | --- |
|  |  |  |  |  |  |

B : **Description and results**

**1. Technical informations**

Probe :_____ Window : close to the tragus 🞏 temporal 🞏other 🞏 ________________

**2. Results :**

Cerebral arteries recorded : R MCA 🞏, R ACA 🞏 L MCA 🞏, L ACA 🞏

|  | Right side | Left side |
| --- | --- | --- |
| Depth (cm) |  |  |
| PSV (cm/sec)/EDV (cm/sec) |  |  |
| RI/PI |  |  |

**Exam considered as normal** 🞏 **abnormal** 🞏 **no opinion** 🞏

**Retained TCD diagnosis  :**

Reverse flow 🞏 Cerebral hypoperfusion 🞏 Intracranial hypertension 🞏 Brain edema 🞏 Asymmetric perfusion 🞏  Hyperhemia 🞏 Others (specify)____________________

**C : TCD’s contribution (within 4 hours after realization)**

**According to you, made TCD a contribution on your managment ? Yes** 🞏 **No** 🞏 **No opinion** 🞏

***If yes***, what kind of contribution? (several possible answers)

- Diagnostic 🞏 :

TCD : confirms 🞏 strengthens 🞏 makes less likely 🞏 excludes 🞏, the main suspected abnormality (see A4)

TCD : strengthens 🞏 makes less likeley 🞏, the suspected neurological diagnosis (see A5)

TCD : confirms 🞏 excludes 🞏 strengthens 🞏 makes less likely 🞏, the suspected diagnosis of intracranial hypertension

Other diagnosis suggested by TCD : Yes 🞏 No 🞏 *If yes,* specify : ______________________________________

- Paraclinical 🞏

Decision of performing supplemental investigations : Yes 🞏 No 🞏

If *yes,* which one?……………………………………………………………………………….

Contraindication for lumbar puncture : Yes 🞏 No 🞏 unapplicable 🞏

- Therapeutic 🞏

Treatment escalation 🞏 Intracranial hypertension treatment 🞏 Neuro-protection 🞏 Intubation 🞏 Hemodynamic optimization 🞏  Treatment deescalation 🞏 Other 🞏, specify : _________________________________________________________________________________

- Monitoring 🞏 : strengthening 🞏 lightening 🞏 continuation 🞏
- Other kind of impact 🞏, specify _____________________________________________________
